# Hipercalemia nas Internações por Insuficiência Cardíaca: Um Contribuinte Subnotificado para Piores Desfechos e Maiores Custos no Brasil

**DOI:** 10.36660/abc.20250394

**Published:** 2026-04-23

**Authors:** Roberto Pecoits-Filho, Pedro Tulio Rocha, Maristela Carvalho da Costa, Pedro Schwartzmann, Lídia Ana Zytynski Moura, Mauricio Longato, Nicolas Segre, Tamisa Rego, Cinthia Montenegro, Thyago Proença de Moraes

**Affiliations:** 1 Arbor Research Collaborative for Health Michigan EUA Arbor Research Collaborative for Health, Michigan – EUA; 2 Pontificia Universidade Católica do Paraná Curitiba PR Brasil Pontificia Universidade Católica do Paraná, Curitiba, PR – Brasil; 3 Universidade Federal do Rio de Janeiro Centro de Ciências da Saúde Rio de Janeiro RJ Brasil Universidade Federal do Rio de Janeiro Centro de Ciências da Saúde, Rio de Janeiro, RJ – Brasil; 4 Hospital São Lucas Rio de Janeiro RJ Brasil Hospital São Lucas, Rio de Janeiro, RJ – Brasil; 5 Hospital das Clínicas da Faculdade de Medicina da Universidade de São Paulo Instituto do Coração São Paulo SP Brasil Instituto do Coração do Hospital das Clínicas da Faculdade de Medicina da Universidade de São Paulo, São Paulo, SP – Brasil; 6 Universidade de São Paulo Faculdade de Medicina Ribeirão Preto SP Brasil Faculdade de Medicina de Ribeirão Preto da Universidade de São Paulo, Ribeirão Preto, SP – Brasil; 7 Nodian São Paulo SP Brasil Nodian, São Paulo, SP – Brasil; 8 AstraZeneca São Paulo SP Brasil AstraZeneca, São Paulo, SP – Brasil

**Keywords:** Insuficiência Cardíaca, Hiperpotassemia, Classificação Internacional de Doenças, Hospitalização

## Abstract

A hipercalemia está associada a um prognóstico ruim na insuficiência cardíaca (IC), mas seu registro em dados administrativos provavelmente é subótimo.

O objetivo foi avaliar a frequência e o impacto da codificação de hipercalemia pela CID durante internações por IC no Brasil.

Analisamos 3.551.738 internações por IC do banco de dados DATASUS (2008–2024). Os códigos da CID foram usados para identificar a hipercalemia. Análises de regressão logística e de Cox avaliaram as associações entre comorbidades, desfechos e utilização de recursos.

Apenas 491 internações (0,014%) foram codificadas para hipercalemia. Esses pacientes eram mais velhos (média de 70,3 vs. 66,6 anos) e apresentavam maiores taxas de doença renal crônica, diabetes e doença cardiovascular. A hipercalemia foi associada ao aumento da mortalidade hospitalar (25% vs. 10%), internações em unidade de terapia intensive (UTI) (17% vs. 11%), diálise (11% vs. 1%) e maior tempo de internação em UTI (9,45 vs. 5,36 dias). Os custos médios foram 59% maiores (2.402 vs. 1.512 BRL).

A hipercalemia é gravemente subnotificada em internações hospitalares por IC no Brasil, apesar de ser um marcador claro de gravidade clínica e maior utilização de recursos. Maior conscientização e melhor codificação podem contribuir para melhores resultados e planejamento.

## Introdução

As hospitalizações por insuficiência cardíaca (IC) continuam sendo um grande desafio clínico e econômico no Brasil. A hipercalemia, frequentemente impulsionada por comorbidades como doença renal crônica (DRC) e diabetes, é um fator de risco independente para desfechos adversos da IC.^1,2^ No entanto, em bancos de dados administrativos como o DATASUS, a hipercalemia pode ser gravemente subnotificada, limitando nossa compreensão de seu impacto.

Este estudo teve como objetivo avaliar a prevalência de codificação de hipercalemia em pacientes hospitalizados por IC no Brasil e avaliar os desfechos clínicos e a utilização de recursos de saúde associados a essa condição, utilizando dados administrativos nacionais.

## Métodos

Incluímos todas as hospitalizações codificadas para IC usando os códigos I50, I50.0, I50.1 e I50.9 da CID-10 no Sistema de Informação Hospitalar DATASUS entre janeiro de 2008 e fevereiro de 2024 (Apêndice). A hipercalemia foi identificada usando o código E875 da CID-10. Comorbidades e eventos clínicos (por exemplo, diabetes, DRC, diálise, lesão renal aguda, infarto agudo do miocárdio e acidente vascular cerebral) foram identificados usando todos os códigos relevantes da CID e de procedimento para maximizar a sensibilidade diagnóstica. As variáveis contínuas foram resumidas como médias e desvios padrão ou medianas e intervalos interquartis, dependendo da distribuição, enquanto as variáveis categóricas foram relatadas como números absolutos e porcentagens. A prevalência de hipercalemia foi calculada dividindo o número de hospitalizações codificadas para hipercalemia pelo número total de hospitalizações por IC. A regressão logística foi usada para avaliar as associações entre hipercalemia e covariáveis clínicas, com os resultados expressos como razões de chances (OR) e intervalos de confiança de 95%. Os desfechos incluíram mortalidade hospitalar, admissão em unidade de terapia intensiva (UTI), necessidade de diálise, tempo de internação e custo da hospitalização. Os desfechos de tempo até o evento (mortalidade em 30 dias e tempo até a alta hospitalar) foram analisados utilizando curvas de Kaplan-Meier, comparados com o teste de log-rank e avaliados posteriormente por meio de modelos de riscos proporcionais de Cox ajustados para fatores de risco estabelecidos. Pacientes hospitalizados por mais de 30 dias foram censurados em ambas as análises de sobrevida.

**Figure:**
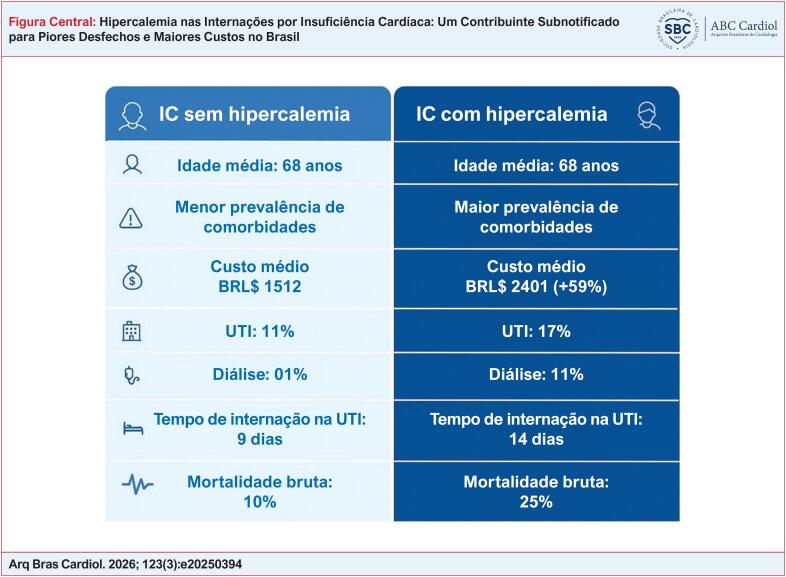
Subnotificação e impacto da hipercalemia em internações por insuficiência cardíaca (Brasil, 2008–2024). IC: insuficiência cardíaca; UTI: unidade de terapia intensiva.

## Resultados

Um total de 3.551.738 internações hospitalares com diagnóstico de IC foram identificadas entre janeiro de 2008 e fevereiro de 2024. Destas, apenas 491 (0,014%) apresentavam um código CID para hipercalemia, sugerindo uma subnotificação significativa. Conforme demonstrado na Tabela 1, os pacientes com hipercalemia eram mais velhos (média de idade de 70,3 anos vs. 66,6 anos) e apresentavam maior prevalência de comorbidades como diabetes, DRC e doença cardiovascular (DCV).

**Tabela 1:** Características da população estratificada pela presença de um relatório de codificação CID para hipercalemia

Variável		Geral	Hipercalemia	Sem hipercalemia
**n**		3.551.738	491	3.551.247
**Idade (anos)**		66,6±16,4	70,3±14,0	66,6±16,4
**Idade > 65 anos**		58,3%	67,4%	58,3%
**Femenina**		48,5%	48,9%	48,5%
**Raça (Branca)**		37,0%	42,8%	37,0%
**Região**				
	Sul	22%	2%	22%
	Sudeste	42%	93%	42%
	Centro-Oeste	7%	1%	7%
	Nordeste	23%	4%	23%
	Norte	5%	0%	5%

Foram evidentes disparidades regionais, com 93% dos casos codificados como hipercalemia concentrados na região Sudeste. Essa distribuição provavelmente reflete não apenas uma variabilidade geográfica real, mas também disparidades nas práticas de codificação dentro do banco de dados nacional (DATASUS). Avaliações anteriores do DATASUS demonstraram heterogeneidade na qualidade e completude da codificação diagnóstica em diferentes regiões brasileiras, frequentemente influenciada pela disponibilidade de recursos, treinamento local e prioridades administrativas. A aparente concentração de casos no Sudeste pode, pelo menos em parte, representar subnotificação em outras regiões, em vez de uma verdadeira diferença epidemiológica, e essa limitação deve ser considerada na interpretação de nossos resultados.

**Figura 1:**
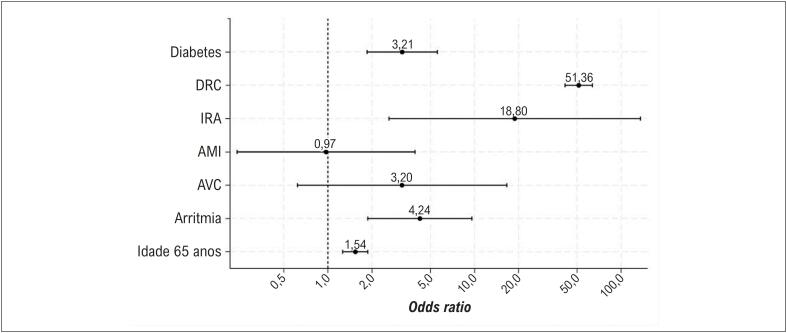
Gráfico de floresta dos fatores de risco associados à hipercalemia. DRC: doença renal crônica; IRA: insuficiência renal aguda; IAM: infarto agudo do miocárdio; AVC: acidente vascular cerebral.

A Figura 1 apresenta um gráfico de floresta da regressão logística, identificando DRC, diabetes, DCV e idade avançada como fortes preditores de codificação de hipercalemia na CID. Esses achados reforçam a associação entre hipercalemia e fatores de risco estabelecidos para IC.

Os desfechos clínicos foram notavelmente piores no grupo com hipercalemia. Conforme detalhado na Tabela 2, a mortalidade hospitalar foi significativamente maior (25% vs. 10%). A admissão na UTI ocorreu em 17% dos pacientes com hipercalemia, em comparação com 11% nos pacientes sem hipercalemia. A proporção de pacientes que necessitaram de diálise foi mais de dez vezes maior (11% vs. 1%).

**Tabela 2:** Eventos de hospitalização e desfechos clínicos de 2008 a 2023

	Hipercalemia	Sem hipercalemia	Valor-p
Dias de internação hospitalar (mediana, IIQ)*	6 (3;12)	4 (IIQ: 3;8)	<0,001
Necessidade de UTI (%)**	17	11	<0,001
Dias na UTI (mediana, IIQ)*	14 (9;25)	9 (IIQ: 9;16)	<0,001
Necessidade de diálise (%)**	11	1	<0,001
Taxa de mortalidade (%)**	25	10	<0,001

* Teste U de Mann-Whitney;

** Teste do qui-quadrado. UTI: unidade de terapia intensiva; IIQ: intervalo interquartil.

**Figura 2:**
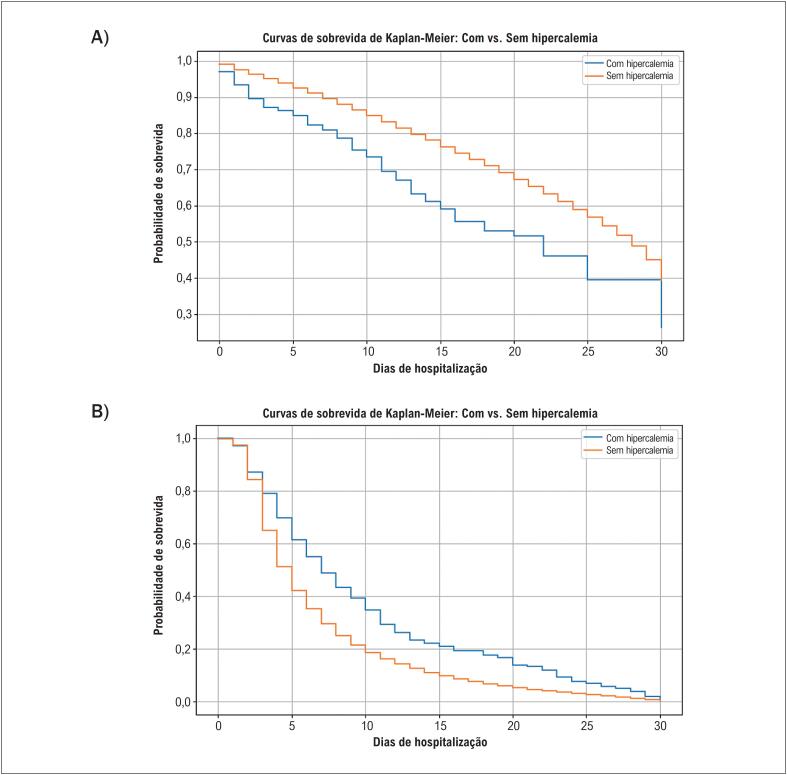
A) Curvas de sobrevida de Kaplan-Meier (ajustadas por Cox) de pacientes com hipercalemia versus aqueles sem hipercalemia. p<0,001. B) Curva de sobrevida de Kaplan-Meier (ajustada por Cox) de pacientes com hipercalemia versus aqueles sem hipercalemia. p<0,001.

A análise de Kaplan-Meier na Figura 2A demonstra uma clara desvantagem de sobrevida em pacientes com hipercalemia. A Figura 2B mostra internações hospitalares prolongadas no grupo com hipercalemia. A mediana do tempo de internação foi de 6 dias (intervalo interquartil [IIQ] 3–12) para pacientes com hipercalemia versus 4 dias (IIQ 3–8) para aqueles sem hipercalemia.

O uso de recursos aumentou significativamente no grupo com hipercalemia. A Tabela 3 mostra que o custo médio de hospitalização foi de 2.401 BRL para pacientes com hipercalemia, 59% maior do que os 1.512 BRL para pacientes sem hipercalemia. Os custos da UTI foram em média de 6.215 BRL, comparados a 3.774 BRL. O tempo de permanência na UTI também foi maior: mediana de 14 dias versus 9 dias.

**Tabela 3:** Análise dos custos de saúde em pacientes com e sem hipercalemia

	Hipercalemia	Sem hipercalemia	Valor-p
**Custo médio (BRL)**	2.401	1.512	< 0,001
**Custo médio da UTI (BRL)**	6.215	3.774	< 0,001

Método estatístico: teste-t.

* BRL: reais brasileiros.

Nossa análise também revelou uma forte associação entre hipercalemia e DRC em estágio terminal, o que provavelmente contribui para os desfechos clínicos desfavoráveis observados. Estudos anteriores relataram taxas de prevalência de hipercalemia superiores a 30% em pacientes com DRC em estágio terminal e quase 15% naqueles em diálise. Assim, o aumento na mortalidade hospitalar e na utilização de recursos de UTI observado entre pacientes com hipercalemia pode refletir, em parte, a gravidade da disfunção renal subjacente, e não apenas a hipercalemia. Isso reforça a necessidade de uma interpretação cautelosa e a importância de se considerar a gravidade da doença renal em análises futuras.^3-5^

Esses resultados, em conjunto, ilustram que, embora a notificação de hipercalemia seja rara, ela identifica um subgrupo de pacientes com IC com risco clínico-econômico e ônus econômico muito maiores (Figura Central). Por fim, a recente introdução do ciclosilicato de sódio e zircônio no Brasil, já aprovado para incorporação no sistema público de saúde, será especialmente importante em pacientes com IC, dado o alto risco de hipercalemia devido à alta prevalência de DRC concomitante e à necessidade de terapias que, embora essenciais para a redução da morbidade e mortalidade, estão associadas ao aumento dos níveis de potássio como efeito adverso.

## Data Availability

Os conteúdos estão disponíveis no link: https://datasus.saude.gov.br/acesso-a-informacao/

## *Material suplementar

*Material suplementar Para informação adicional, por favor, clique aqui.
