# Supplementary material for: Hipercalemia nas Internações por Insuficiência Cardíaca: Um Contribuinte Subnotificado para Piores Desfechos e Maiores Custos no Brasil
Source: Arq Bras Cardiol. 2026 Apr 23;123(3):e20250394. [Article in Portuguese] doi: 10.36660/abc.20250394 (PMC13128216; doi:10.36660/abc.20250394)
Supplement: *Material suplementar [file 0066-782x-abc-123-3-e20250394-Supp01.pdf]

Appendix: ICD code details

Code	Description
**Dialysis**	
305010069	Hemodialysis I (maximum 3 sessions per week)
305010123	Hemodialysis for patients with positive serology for HIV and/or Hepatitis B and/or Hepatitis C (exceptional case - maximum 1 session per week)
305010026	Intermittent peritoneal dialysis (IPD) (maximum 2 sessions per week)
305010034	Peritoneal dialysis for acute renal patients
305010107	Hemodialysis II (maximum 3 sessions per week)
301130060	Multidisciplinary follow-up for CKD stage 05 pre-dialysis
305010204	Pediatric hemodialysis (maximum 4 sessions per week)
305010042	Continuous hemodialysis
305010018	Intermittent peritoneal dialysis (IPD) (1 session per week - exceptional case)
305010220	Additional payment for hemodialysis sessions for patients with suspected or confirmed COVID-19 (maximum 4 sessions per week)
305010077	Hemodialysis I for HIV-positive patients (exceptional case - maximum 1 session per week)
305010093	Hemodialysis II (maximum 1 session per week - exceptional case)

305010085	Hemodialysis I for HIV-positive patients (maximum 3 sessions per week)
305010115	Hemodialysis for patients with positive serology for HIV and/or Hepatitis B and/or Hepatitis C (maximum 3 sessions per week)
301130051	Multidisciplinary follow-up for CKD stage 04 pre-dialysis
305010050	Hemodialysis I (maximum 1 session per week - exceptional case)
305010131	Hemodialysis for acute/chronic renal patients without initiated dialysis treatment
**Pregnancy**	
0310010039	Normal delivery
0411010034	Cesarean delivery
0310010047	Normal delivery in high-risk pregnancy
0411010026	Cesarean delivery in high-risk pregnancy
0411010042	Cesarean delivery with tubal ligation
**Diabetes**	
E10	Insulin-dependent diabetes mellitus
E10.0	Insulin-dependent diabetes mellitus – with coma
E10.1	Insulin-dependent diabetes mellitus – with ketoacidosis
E10.2	Insulin-dependent diabetes mellitus – with renal complications
E10.3	Insulin-dependent diabetes mellitus – with ophthalmological complications

E10.4	Insulin-dependent diabetes mellitus – with neurological complications
E10.5	Insulin-dependent diabetes mellitus – with peripheral circulatory complications
E10.6	Insulin-dependent diabetes mellitus – with specified complications
E10.7	Insulin-dependent diabetes mellitus – with multiple complications
E10.8	Insulin-dependent diabetes mellitus – with unspecified complications
E10.9	Insulin-dependent diabetes mellitus – without complications
**Chronic Kidney Disease**	
N18	Chronic renal failure
N18.2	Chronic kidney disease, stage 2
N18.3	Chronic kidney disease, stage 3
N18.4	Chronic kidney disease, stage 4
N18.5	Chronic kidney disease, stage 5
N18.9	Chronic kidney disease, unspecified
**AKI**	
N17.0	Acute renal failure with tubular necrosis
N17.1	Acute renal failure with cortical necrosis
N17.2	Acute renal failure with medullary necrosis

**Acute Miocardial Infarction**	
I21	Acute myocardial infarction
I21.0	Acute transmural myocardial infarction of the anterior wall
I21.1	Acute transmural myocardial infarction of the inferior wall
I21.2	Acute transmural myocardial infarction in other locations
I21.3	Acute transmural myocardial infarction, unspecified location
I21.4	Acute subendocardial myocardial infarction
I21.9	Unspecified acute myocardial infarction
**Subarachnoid hemorrhage**	
I60	Subarachnoid hemorrhage from the siphon and bifurcation of the carotid artery
I60.1	Subarachnoid hemorrhage from the middle cerebral artery
I60.2	Subarachnoid hemorrhage from the anterior communicating artery
I60.3	Subarachnoid hemorrhage from the posterior communicating artery
I60.4	Subarachnoid hemorrhage from the basilar artery
I60.5	Subarachnoid hemorrhage from the vertebral artery
I60.6	Subarachnoid hemorrhage from other intracranial arteries
I60.7	Subarachnoid hemorrhage from an unspecified intracranial artery

I60.8	Other subarachnoid hemorrhages
I60.9	Unspecified subarachnoid hemorrhage
**Intracerebral hemorrhage**	
I61	Intracerebral hemorrhage
I61.1	Subcortical hemispheric intracerebral hemorrhage
I61.2	Cortical hemispheric intracerebral hemorrhage
I61.3	Unspecified hemispheric intracerebral hemorrhage
I61.4	Brainstem intracerebral hemorrhage
I61.5	Cerebellar intracerebral hemorrhage
I61.6	Intraventricular intracerebral hemorrhage
I61.7	Intracerebral hemorrhage in multiple locations
I61.8	Other intracerebral hemorrhages
I61.9	Unspecified intracerebral hemorrhage
**Stroke**	
I64	Stroke, unspecified as hemorrhagic or ischemic
**Retinal vascular occlusions**	
H34	Retinal vascular occlusions
H34.0	Transient retinal arterial occlusion
H34.1	Central retinal artery occlusion
H34.2	Other retinal artery occlusions
H34.8	Other retinal vascular occlusions

H34.9	Unspecified retinal vascular occlusion
**Others**	
G45	Transient ischemic cerebral attacks and related syndromes
G45.0	Vertebrobasilar artery syndrome
G45.1	Carotid artery (hemispheric) syndrome
G45.2	Multiple and bilateral precerebral artery syndrome
G45.3	Transient monocular blindness (amaurosis fugax)
G45.4	Transient global amnesia
G45.8	Other transient ischemic cerebral attacks and related syndromes
G45.9	Unspecified transient cerebral ischemia
**Hyperkalemia**	
E875	Hyperkalemia
**Arrhythmia**	
I49.0	Ventricular flutter and fibrillation
I49.1	Premature atrial depolarization
I49.2	Premature junctional depolarization
I49.3	Premature ventricular depolarization
I49.9	Unspecified cardiac arrhythmia
**Cerebral infarction**	
I63	Cerebral infarction

I63.0	Cerebral infarction due to thrombosis of precerebral arteries
I63.1	Cerebral infarction due to embolism of precerebral arteries
I63.2	Cerebral infarction due to unspecified occlusion or stenosis of precerebral arteries
I63.3	Cerebral infarction due to thrombosis of cerebral arteries
I63.4	Cerebral infarction due to embolism of cerebral arteries
I63.5	Cerebral infarction due to unspecified occlusion or stenosis of cerebral arteries
I63.8	Other cerebral infarctions
I63.9	Unspecified cerebral infarction
