# Supplementary material for: Hipercalemia nas Internações por Insuficiência Cardíaca: Um Contribuinte Subnotificado para Piores Desfechos e Maiores Custos no Brasil
Source: Arq Bras Cardiol. 2026 Apr 23;123(3):e20250394. [Article in Portuguese] doi: 10.36660/abc.20250394 (PMC13128216; doi:10.36660/abc.20250394)
Supplement: Translation

# Translation: Hyperkalemia in Heart Failure Hospitalizations: An Underreported Contributor to Poor Outcomes and Higher Costs in Brazil

**DOI:** 10.36660/abc.20250394i

**Authors:** Roberto Pecoits-Filho, Pedro Tulio Rocha, Maristela Carvalho da Costa, Pedro Schwartzmann, Lídia Ana Zytynski Moura, Mauricio Longato, Nicolas Segre, Tamisa Rego, Cinthia Montenegro, Thyago Proença de Moraes

**Affiliations:** 1 Michigan USA Arbor Research Collaborative for Health, Michigan – USA; 2 Curitiba PR Brazil Pontificia Universidade Católica do Paraná, Curitiba, PR – Brazil; 3 Rio de Janeiro RJ Brazil Universidade Federal do Rio de Janeiro Centro de Ciências da Saúde, Rio de Janeiro, RJ – Brazil; 4 Rio de Janeiro RJ Brazil Hospital São Lucas, Rio de Janeiro, RJ – Brazil; 5 São Paulo SP Brazil Instituto do Coração do Hospital das Clínicas da Faculdade de Medicina da Universidade de São Paulo, São Paulo, SP – Brazil; 6 Ribeirão Preto SP Brazil Faculdade de Medicina de Ribeirão Preto da Universidade de São Paulo, Ribeirão Preto, SP – Brazil; 7 São Paulo SP Brazil Nodian, São Paulo, SP – Brazil; 8 São Paulo SP Brazil AstraZeneca, São Paulo, SP – Brazil

**Keywords:** Heart Failure, Hyperkalemia, International Classification of Diseases, Hospitalization

## Abstract

Hyperkalemia is associated with poor prognosis in heart failure (HF), but its reporting in administrative data is likely suboptimal.

The aim was to evaluate the frequency and impact of hyperkalemia ICD coding during HF hospitalizations in Brazil.

We analyzed 3,551,738 HF hospitalizations from the DATASUS database (2008–2024). ICD codes were used to identify hyperkalemia. Logistic and Cox regression analyses assessed associations between comorbidities, outcomes, and resource utilization.

Only 491 hospitalizations (0.014%) were coded for hyperkalemia. These patients were older (mean 70.3 vs. 66.6 years) and had higher rates of chronic kidney disease, diabetes, and cardiovascular disease. Hyperkalemia was associated with increased in-hospital mortality (25% vs. 10%), ICU admissions (17% vs. 11%), dialysis (11% vs. 1%), and longer ICU stays (9.45 vs. 5.36 days). Average costs were 59% higher (2,402 vs. 1,512 BRL).

Hyperkalemia is severely underreported in Brazilian HF hospitalizations, despite being a clear marker of clinical severity and higher resource use. Improved awareness and coding may support better outcomes and planning.

## Introduction

Heart failure (HF) hospitalizations remain a major clinical and economic challenge in Brazil. Hyperkalemia, often driven by comorbid conditions such as chronic kidney disease (CKD) and diabetes, is an independent risk factor for adverse HF outcomes.^1,2^ However, in administrative datasets like DATASUS, hyperkalemia may be severely underreported, limiting our understanding of its impact.

This study aimed to assess the prevalence of hyperkalemia coding among patients hospitalized for HF in Brazil and to evaluate the clinical outcomes and healthcare resource utilization associated with this condition using national administrative data.

## Methods

We included all hospitalizations coded for HF using ICD-10 codes I50, I50.0, I50.1, and I50.9 in the DATASUS Hospital Information System between January 2008 and February 2024 (Appendix). Hyperkalemia was identified using the ICD-10 code E875. Comorbidities and clinical events (e.g., diabetes, CKD, dialysis, acute kidney injury, acute myocardial infarction, and stroke) were identified using all relevant ICD and procedure codes to maximize diagnostic sensitivity. Continuous variables were summarized as means and standard deviations or medians and interquartile ranges, depending on distribution, while categorical variables were reported as absolute numbers and percentages. The prevalence of hyperkalemia was calculated by dividing the number of hyperkalemia-coded hospitalizations by the total number of HF hospitalizations. Logistic regression was used to evaluate associations between hyperkalemia and clinical covariates, with results expressed as odds ratios (OR) and 95% confidence intervals). Outcomes included in-hospital mortality, ICU admission, dialysis requirement, length of stay, and hospitalization cost. Time-to-event outcomes (30-day mortality and time to hospital discharge) were analyzed using Kaplan–Meier curves, compared with the log-rank test, and further assessed using Cox proportional hazards models adjusted for established risk factors. Patients hospitalized for more than 30 days were censored in both survival analyses.

## Results

A total of 3,551,738 hospitalizations with a HF diagnosis were identified from January 2008 to February 2024. Of these, only 491 (0.014%) had an ICD code for hyperkalemia, suggesting substantial underreporting. As shown in Table 1, patients with hyperkalemia were older (mean age 70.3 vs. 66.6 years) and had a higher prevalence of comorbidities such as diabetes, CKD and cardiovascular disease (CVD).

**Table 1:** Characteristics of the population stratified by the presence of an ICD coding report for hyperkalemia

Variable		Overall	Hyperkalemia	No hyperkalemia
**n**		3,551,738	491	3,551,247
**Age (years)**		66.6±16.4	70.3±14.0	66.6±16.4
**Age > 65 years**		58.3%	67.4%	58.3%
**Female**		48.5%	48.9%	48.5%
**Race (White)**		37.0%	42.8%	37.0%
**Region**				
	South	22%	2%	22%
	Southeast	42%	93%	42%
	Midwest	7%	1%	7%
	Northeast	23%	4%	23%
	North	5%	0%	5%

**Figure:** Underreporting and Impact of Hyperkalemia in Heart Failure Hospitalizations (Brazil, 2008–2024).

Regional disparities were evident, with 93% of hyperkalemia-coded cases concentrated in the Southeast region. This distribution likely reflects not only a true geographic variability but also disparities in coding practices within the national database (DATASUS). Previous evaluations of DATASUS have demonstrated heterogeneity in the quality and completeness of diagnostic coding across different Brazilian regions, often influenced by resource availability, local training, and administrative priorities. The apparent concentration of cases in the Southeast may, at least in part, represent underreporting in other regions rather than a true epidemiological difference, and this limitation must be considered when interpreting our results.

Figure 1 displays a forest plot from logistic regression, identifying CKD, diabetes, CVD, and older age as strong predictors of hyperkalemia ICD coding. These findings reinforce the association between hyperkalemia and established HF risk factors.

**Figure 1:** Forest plot of the risk factors associated with hyperkalemia. CKD: chronic kidney disease; AKI: acute kidney injury; AMI: acute myocardial infarction.

Clinical outcomes were notably worse in the hyperkalemia group. As detailed in Table 2, in-hospital mortality was significantly higher (25% vs. 10%). ICU admission occurred in 17% of hyperkalemic patients compared to 11% in non-hyperkalemic patients. The proportion requiring dialysis was over tenfold higher (11% vs. 1%).

**Table 2:** Hospitalization events and clinical outcomes from 2008 to 2023

	Hyperkalemia	No Hyperkalemia	p-value
Days in the hospital stay (median, IQR)*	6 (3;12)	4 (IQR: 3;8)	<0.001
Need for ICU (%)**	17	11	<0.001
Days in the ICU (median, IQR)*	14 (9;25)	9 (IQR: 9;16)	<0.001
Need for dialysis (%)**	11	1	<0.001
Mortality rate (%)**	25	10	<0.001

* Mann–Whitney U test;

** Chi-square test. HK: hyperkalemia; ICU: intensive care unit; IQR: interquartile range

Kaplan-Meier analysis in Figure 2A demonstrates a clear survival disadvantage in hyperkalemic patients. Figure 2B shows prolonged hospital stays in the hyperkalemia group. Median length of stay was 6 days (IQR 3–12) for hyperkalemic patients versus 4 days (IQR 3–8) for those without hyperkalemia.

**Figure 2:** A) Kaplan-Meier survival curves (Cox adjusted) of patients with hyperkalemia versus those without. p<0.001. B) Kaplan-Meier length of hospitalization (Cox adjusted) of patients with hyperkalemia versus those without.

Resource use was markedly increased in the hyperkalemia group. Table 3 shows that the mean hospitalization cost was 2,401 BRL for hyperkalemic patients, 59% higher than the 1,512 BRL for patients without hyperkalemia. ICU costs averaged 6,215 BRL compared to 3,774 BRL. ICU length of stay was also longer: median 14 days versus 9 days.

**Table 3:** Analysis of Healthcare Costs in Patients With Versus Without

	Hyperkalemia	No Hyperkalemia	p-value
**Mean cost(BRL)**	2,401	1,512	< 0.001
**Mean cost ICU (BRL)**	6,215	3,774	< 0.001

Statistical method: t-test.

* BRL: Brazilian reais.

Our analysis also revealed a strong association between hyperkalemia and ESKD, which likely contributes to the poor clinical outcomes observed. Previous studies have reported hyperkalemia prevalence rates exceeding 30% in patients with ESKD and nearly 15% in those undergoing dialysis. As such, the increase in in-hospital mortality and ICU resource use seen among hyperkalemia-coded patients may partly reflect the severity of underlying kidney dysfunction rather than hyperkalemia alone. This underscores the need for cautious interpretation and the importance of accounting for renal disease severity in future analyses.^3-5^

These results collectively illustrate that while hyperkalemia reporting is rare, it identifies a subgroup of HF patients at much higher clinical economic risk and economic burden (Central Illustration). Finally, the recent introduction of sodium zirconium cyclosilicate in Brazil, already approved for incorporation into the public health system, will be especially important in patients with HF, given their high risk of hyperkalemia due to a high prevalence of concomitant CKD and the need for therapies that, while essential for reducing morbidity and mortality, are associated with increased potassium levels as an adverse effect.

## Data Availability

The content is available at the link: https://datasus.saude.gov.br/acesso-a-informacao/

## *Supplemental Materials

*Supplemental Materials For additional information, please click here.
